# Tufted angioma (Angioblastoma) of eyelid in adults-report of two cases

**DOI:** 10.1186/1746-1596-8-153

**Published:** 2013-09-17

**Authors:** Ruchi Mittal, Devjyoti Tripathy

**Affiliations:** 1 Dalmia Ophthalmic Pathology Services, L.V. Prasad Eye Institute, Bhubaneswar, Orissa, India; 2 Department of Ophthalmic Plastic Surgery, Orbit and Ocular Oncology, L.V. Prasad Eye Institute, Bhubaneswar, Orissa, India

**Keywords:** Tufted angioma, Angioblastoma, Eyelid, Adults, Kasabach-Merritt syndrome

## Abstract

**Abstract:**

Tufted angioma, first recognized in Japanese literature as “Angioblastoma of Nagakawa”, is a rare benign vascular tumour with a variable clinical presentation. It commonly manifests as a macule, papule or nodule in infancy or childhood in the region of the upper trunk and neck. Here in we report two cases of this rare progressive angioma as lesions of the eyelid in adults. Tufted angioma has a classical “cannon ball” like appearance of vascular tufts on histopathology. Immunohistochemical staining with actin highlights the spindly stromal cells surrounding the capillaries. Complete physical examination and haematological work up is recommended in patients with tufted angioma to exclude rare association of port wine stain and Kasabach-Merritt syndrome with this rare entity. To the best of our knowledge, our cases illustrate the first case report of tufted angioma presenting as an eyelid lesion.

**Virtual Slides:**

The virtual slide(s) for this article can be found here: http://www.diagnosticpathology.diagnomx.eu/vs/1230909536950947.

## Background

Tufted angioma, first recognized in Japanese literature as “Angioblastoma of Nagakawa”, is a benign progressive angioma, with a variable clinical presentation [1]. Wilson-Jones and Orkin [2] coined the term “tufted angioma” for this unusual pattern of angiomatous proliferation which was found to have some morphological similarities with strawberry nevi. However, as the name suggests, the lesion was seen as cannon ball like, small circumscribed angiomatous tufts and nodules in the dermis and subcutaneous tissue with characteristic lymphangioma-like vessels [3]. Tufted angioma (TA) can be congenital or acquired, commonly presents in infancy or early childhood, can be present at birth in approximately 25% of cases [4], and few cases of TA have been reported in adults [5]. It commonly presents as a macule, papule or plaque over the upper trunk, neck and proximal part of the limbs [6], however involvement of other locations like face, oral mucosa and lip [5] is also known. A pubmed search of dermatological and ophthalmic literature using key words “Tufted angioma, Angioblastoma, eyelid, adults, Kasabach-Merritt syndrome” did not reveal any case of tufted angioma presenting as an eyelid lesion. To the best of our knowledge, involvement of the eyelid with TA is previously unreported. Herein, we report 2 cases of TA of eyelid in adult patients which were clinically diagnosed as lymphangioma and epidermal cyst. This is a retrospective study, approved by Institutional review board of L.V. Prasad Eye Institute as a retrospective study.

## Case presentation

### Case 1

A 17-year-old male presented with a history of a gradually progressive, painless swelling of right lower eyelid of 6 years’ duration. There was no history of trauma, previous ocular surgery or presence of similar lesion elsewhere in the body. Clinical examination revealed a right lower eyelid soft tissue mass not fixed to the underlying tarsus. It was non-tender and spongy to firm on palpation. On eversion of the eyelid the lower palpebral conjunctiva was unremarkable. Rest of the ocular examination was normal. A vascular lesion, probably a lymphangioma, was suspected. Systemic examination and complete blood picture was normal and a biopsy was planned. At biopsy under local anesthesia, the mass appeared o be reddish and ill-defined with intermingling soft and firm areas. Piecemeal excision of the lesion was done and the excised tissue was submitted for histological examination.

### Case 2

A 44-year-old female presented with a slowly progressive painless swelling of the right upper eyelid of about 3 years’ duration. There was no other significant ocular or systemic history. Clinical examination revealed a mobile, non-tender, and cystic to firm nodular mass, not fixed to the tarsus. Systemic evaluation and complete blood picture was normal. Clinically, the lesion was thought to be an epidermal cyst. At biopsy under local anesthesia, a nodular, well delineated, non-encapsulated, firm mass about 15 mm in its maximum dimension was excised in Toto and submitted for histological evaluation.

**Figure 1:**
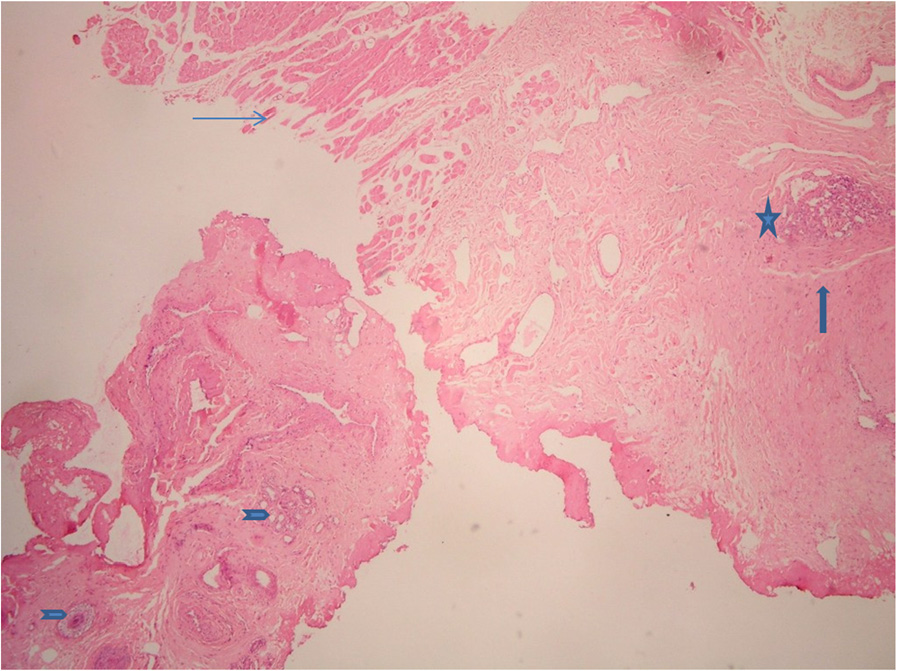
**(Case 1) Low magnification of eyelid stromal tissue with angiomatous tufts of tufted angioma of eyelid.** Eyelid stroma with adnexal tissues (arrow head marked), striated muscle bundles of orbicularis oculi (thin arrow marked), with cannon ball appearance of vascular tufts (asterix marked) surrounded by dilated lymphatic like spaces (thick arrow marked), (stain, haematoxylin and eosin; original magnification, ×40).

**Figure 2:**
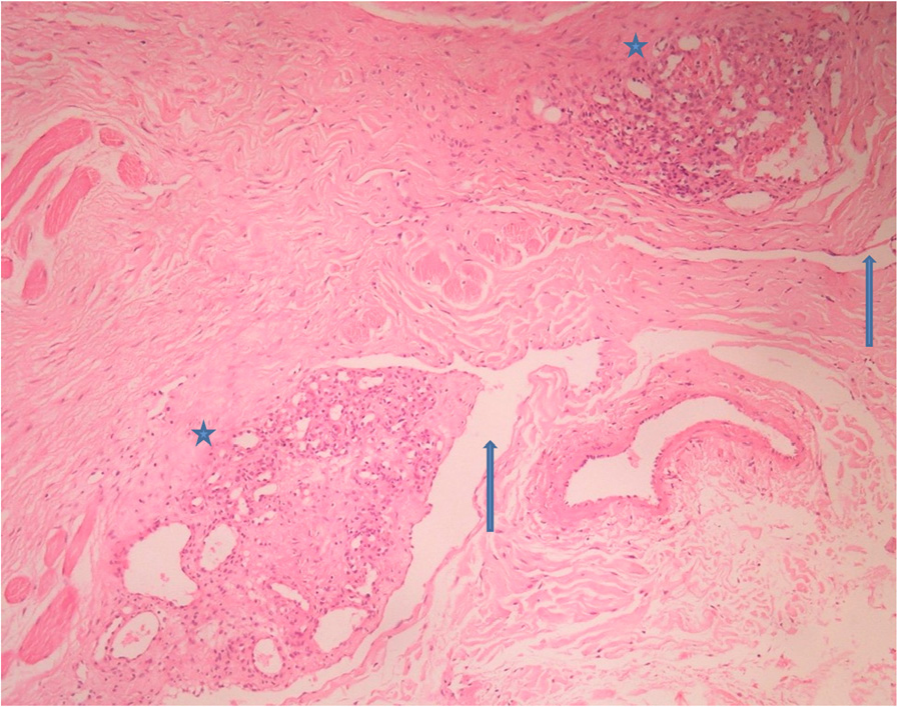
**(Case 1) Cannon-ball like arrangement of vascular tufts in tufted angioma.** Circumscribed ovoid cannon-ball like arrangement of vascular tufts scattered in the eyelid stroma (asterix marked), surrounded by dilated lymphatic like spaces (arrow marked). Striated muscle bundles of orbicularis oculi are also noted in the left of the photomicrograph, (stain, haematoxylin and eosin; original magnification, × 100).

Histopathology sections from case 1 showed multiple fragments of stromal tissue of eyelid with adnexal structures, striated muscle bundles of orbicularis oculi (Figure 1) and relatively circumscribed ovoid foci of closely set capillaries scattered throughout the stroma (Figures 1 and 2). Capillaries were predominantly bloodless, lined by plump endothelial cells and surrounded by prominent oval to slightly spindly cells. Dilated lymphatic–like vessels, some of which were crescent shaped, were seen in close approximation to, or surrounding the capillary aggregates. Cellular atypia or mitoses were absent in multiple serial sections studied.

**Figure 3:**
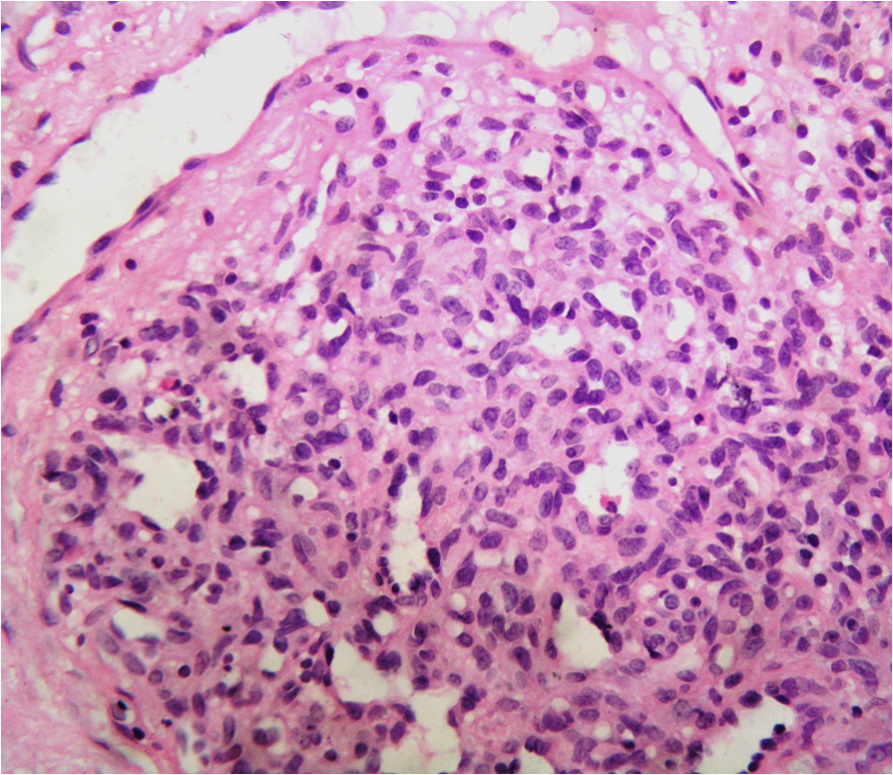
**(Case 2) Angiomatous tissue composed of endothelial cell and predominant population of pericytes.** Circumscribed aggregate of angiomatous tissue composed of bloodless capillaries lined by plump endothelial cells, surrounded by bland oval to slightly spindly pericytes. Dilated lymphatic like space is seen in close approximation to the vascular tufts, (stain, haematoxylin & eosin; original magnification, ×400).

Histopathology sections from case 2 showed a single fragment of stromal tissue of eyelid with striated muscle bundles of orbicularis oculi and closely set relatively circumscribed ovoid to round angiomatous aggregates. These angiomatous aggregates were composed of blood less capillaries with slit like or mildly dilated lumina, lined by plump bland endothelial cells (Figure 3). These capillaries were surrounded by short spindly to oval bland cells (Figure 3). Dilated lymphatic like vascular spaces were seen in close approximation to the angiomatous tufts. No mitotic figure was identified in the sections studied.

**Figure 4:**
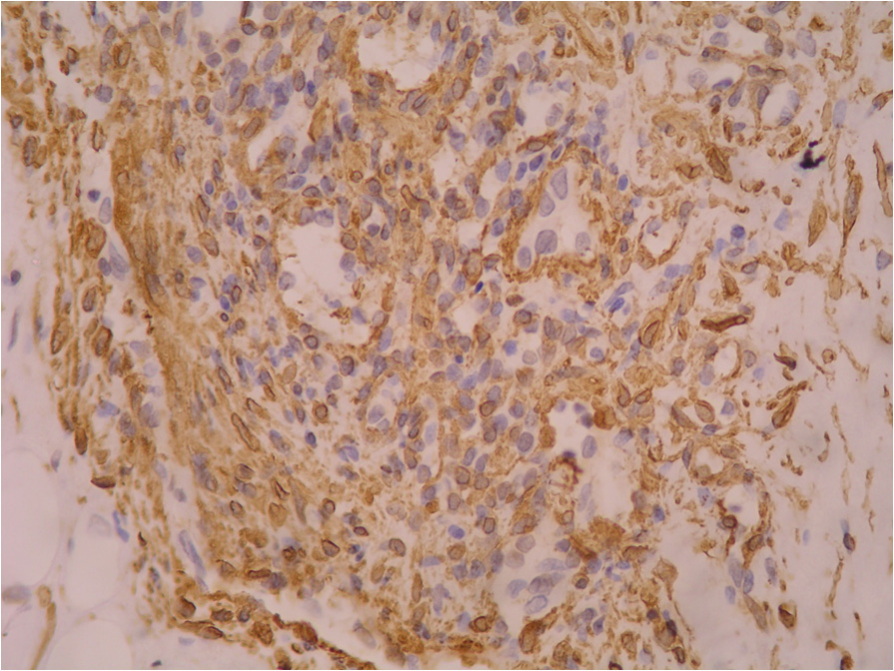
**Immunohistochemical staining of pericytes in tufted angioma with Actin.** Actin is seen decorating the oval to spindly cells (×400).

**Figure 5:**
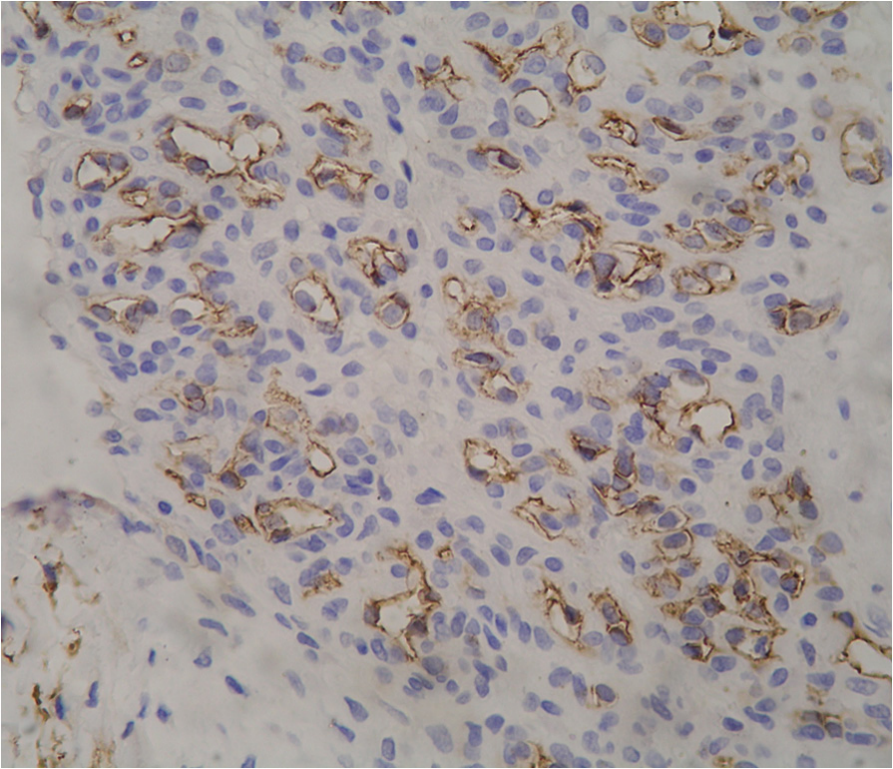
**Immunohistochemical staining of endothelial cells in tufted angioma with CD34.** CD34 is seen highlighting the plump endothelial cells (×400).

Immunohistochemical staining in both cases showed similar findings. Staining with actin revealed a prominent pericytic component (Figure 4). CD34 stained the endothelial cells (Figure 5). Ki-67 showed a very low proliferation index. A diagnosis of tufted angioma of the eyelid was made in both the cases based on the above histopathological and immunohistochemical findings.

Complete physical examination by a dermatologist was normal. Both cases have not had a recurrence in over 12 months of follow up.

## Discussion

Tufted angioma is a rare, slowly progressive benign vascular tumor which can have variable clinical morphology. It can present as red or bluish to violaceous papule, plaque or nodule over neck, upper back and proximal part of limbs typically in childhood and infancy. These lesions may also develop in adults or the elderly [7]. There is no sex predilection [8]. The lesions range from few millimeters to few centimeters in size but may be extensive covering larger areas and can be multifocal [9]. Most of the lesions are asymptomatic but may present with tenderness, hypertrichosis and hyperhidrosis [8,10,11]. Tenderness, hypertrichosis, and induration can be useful in differentiating TA from common hemangioma [12]. Microscopically TA has a classical morphology. It is found dispersed in the dermis and occasionally extends into the subcutaneous tissue and is seen as discrete round to ovoid angiomatous aggregates [8]. These aggregates are composed of relatively bloodless, poorly canalized capillaries. Capillaries are lined by plump endothelial cells, which may show slight spindling. The endothelial cells show reactivity for several markers, including CD31, CD34 and von Willebrand factor (factor VIII). In our case, CD34 was used to highlight the endothelial cells. Pericytes are seen surrounding the capillaries and are the predominant cellular component of TA. These cells have indistinct cell boundaries, scant cytoplasm, and oval to slightly elongated nuclei with bland morphology. Dilated crescent shaped lymphatic like vascular channels are seen surrounding the angiomatous lobules. These channels are lined by plump to flattened cells with oval to slightly spindly nuclei.

Tufted angioma in childhood needs differentiation from strawberry nevi and kaposiform hemangioendothelioma [4]. Angiomatous aggregates of strawberry nevi are more massive and replace wider planes. Eyelid lesions tend to have involvement of deeper orbital structures. Kaposiform hemangioendothelioma is morphologically intermediate between strawberry nevi and Kaposi sarcoma [8], is more commonly seen in childhood and shows capillaries with lobular pattern that are locally infiltrative. Periphery of the tumor shows micro thrombi within the capillaries. Angioblastoma of Nagakawa (TA) should not be confused with the term giant cell Angioblastoma (GCA), which is a rare hemangioma-like lesion of infancy [13]. GCA is characterized by nodular, linear and plexiform granuloma–like aggregates of histiocytes–like cells and giant cells, surrounding capillary sized vessels lined by plump endothelial cells. Background is loose, mesenchymal with mononuclear inflammatory cell infiltrate and mast cells [13,14]. In adults, TA needs to be differentiated from Kaposi’s sarcoma and low grade angiosarcoma [2,7]. Endothelial cells in TA appear plump to slightly spindled and lack the spindling of Kaposi’s sarcoma [8]. Vascular spaces of TA are relatively bloodless when compared to blood filled spaces in Kaposi’s sarcoma. Lack of nuclear atypia excludes angiosarcoma [7]. Other diagnostic hints of angiosarcoma include presence of sinusoid like spaces, dissection of collagen, mitoses, necrosis and haemorrhage [8,15]. Cases with clinical suspicion of TA should be worked up with complete physical examination and complete blood count with red cell morphology to exclude consumptive coagulopathy and Kasabach–Merritt syndrome (KMS) [16,17]. Prothrombin time and activated tpartial thromboplastin time should be performed in patients with thrombocytopenia. Rare association of Port wine stain with TA is also reported [5,17]. Spontaneous regression is known, but is exceptionally rare and regression may occur over a period varying from months to years [18,19].

## Conclusions

As evident by this paper, TA although not described in the eyelid before, should be considered as a differential diagnosis of benign eyelid vascular lesions. TA can have variable clinical presentation and can present at any age. They can be ill defined to circumscribed, and should morphologically be distinguished from other commoner benign and malignant vascular eyelid lesions. A complete physical examination and hematological work up has been recommended in patients with classical TA to exclude rare association of Port wine stain and KMS. Whether a significant risk of the KMS exists in patients of eyelid TA is not currently known. Considering the smaller size of eyelid lesions this risk is less likely. Nonetheless it may be prudent to get a detailed physical and hematological work up done in all cases diagnosed with eyelid TA.

## Consent

Written informed consent was obtained from the patient for publication of this Case Report and accompanying images. A copy of the written consent is available for review by the Editor-in-chief of this journal.

## Abbreviations

TA: Tufted angioma; KMS: Kasabach-Merritt syndrome.

## Competing interests

The authors declare that they have no competing interests.

## Authors’ contributions

RM carried out the histological and immunohistochemical work up, compiled the cases and drafted the manuscript. DT carried out the clinical work up, provided the clinical information, participated in designing the manuscript and edited the manuscript. Both the authors read and approved the final manuscript.
